# Enhanced detection and comprehensive *in situ* phenotypic characterization of circulating and disseminated heteroploid epithelial and glioma tumor cells

**DOI:** 10.18632/oncotarget.4819

**Published:** 2015-07-29

**Authors:** Feng Ge, Haishi Zhang, Daisy Dandan Wang, Linda Li, Peter Ping Lin

**Affiliations:** ^1^ Department of Thoracic Surgery, Capital Medical University School of Oncology and Chaoyang Hospital, Beijing, China; ^2^ Department of Neurosurgery, Huashan Hospital, Fudan University, Shanghai, China; ^3^ Cytelligen, San Diego, California, USA

**Keywords:** CTC and DTC subtypes, iFISH, subtraction enrichment, cytokeratin (CK) 18, *in situ* phenotyping and karyotyping

## Abstract

Conventional strategy of anti-EpCAM capture and immunostaining of cytokeratins (CKs) to detect circulating tumor cells (CTCs) is limited by highly heterogeneous and dynamic expression or absence of EpCAM and/or CKs in CTCs. In this study, a novel integrated cellular and molecular approach of subtraction enrichment (SE) and immunostaining-FISH (iFISH) was successfully developed. Both large or small size CTCs and circulating tumor microemboli (CTM) in various biofluid samples including cerebrospinal fluid (CSF) of cancer patients and patient-derived-xenograft (PDX) mouse models were efficiently enriched and comprehensively identified and characterized by SE-iFISH. Non-hematopoietic CTCs with heteroploid chromosome 8 were detected in 87–92% of lung, esophageal and gastric cancer patients. Characterization of CTCs performed by CK18-iFISH showed that CK18, the dual epithelial marker and tumor biomarker, was strong positive in only 14% of lung and 24% of esophageal CTCs, respectively. Unlike conventional methodologies restricted only to the large and/or both EpCAM and CK positive CTCs, SE-iFISH enables efficient enrichment and performing *in situ* phenotypic and karyotypic identification and characterization of the highly heterogeneous CTC subtypes classified by both chromosome ploidy and the expression of various tumor biomarkers. Each CTC subtype may possess distinct clinical significance relative to tumor metastasis, relapse, therapeutic drug sensitivity or resistance, etc.

## INTRODUCTION

The clinical implications of circulating tumor cells (CTCs) have been reported elsewhere [1, 2]. The American Society of Clinical Oncology (ASCO) has recently accepted quantification of CTC as a novel breast cancer biomarker [3].

Anti-EpCAM-dependent antibody capture [1] and tumor cell size-based filtration [4] currently constitute common strategies for isolating CTCs [5–7]. However, increasing evidence has emerged that besides existence of significant amount of small size CTCs, clinical application of anti-EpCAM strategy is significantly limited due to inherent methodological deficiencies and intrinsic heterogeneity of cell biomarkers in cancer patients. Anti-EpCAM-dependent capture of CTCs is based upon recognition and binding of solid phase (such as magnetic particle or microfludics)-conjugated antibody against epithelial cell surface adhesion molecule EpCAM [1]. Although EpCAM is expressed on many types of epithelial tumor cells, it is highly heterogeneously and dynamically expressed [8] or even absent [9] on cells of several types of cancer, such as melanoma, glioma and mesenchymal tumors [10]. It has been reported that only 70% of the examined 134 epithelial solid tumors express EpCAM [11], and there is a significant phenotypic heterogeneity of dynamically expressed EpCAM even among the individual CTC within the same sample [8, 9]. Interestingly, it has been recently published that CTCs may lose EpCAM during epithelial-mesenchymal transition (EMT) [8, 9], and only EpCAM-negative CTCs (such as breast cancer CTCs) possess enhanced potential to metastasize to the brain [12], suggesting expression of EpCAM may be down-regulated or absent in association with cancer progression and metastasis [12, 13]. This is likely to result in failure to capture CTCs with EpCAM-dependent strategies [8, 14]. In addition, because intracellular signaling pathways of neoplastic cells are activated by crosslinking of cell surface molecules (such as EpCAM) following antibody binding [8, 15–17], it is not surprising that subsequent analyses of intracellular signaling events in CTCs isolated by anti-EpCAM may result in post-collection artifacts in the CTCs perturbed by anti-EpCAM.

The biological and clinical significance of cytokeratin 18 (CK18) expression by numerous carcinomas has been reported [18]. Post-translational modification, up- or down-regulation of tumor biomarkers, such as CK18 protein in tumor cells revealed and quantified by phenotypic immunostaining, correlates with cancer progression [19], cell migration [20], and differentiation in hepatocellular carcinoma (HCC) [21], as well as with staging, metastasis and recurrence in esophageal squamous cell, renal cell, breast, and nasopharyngeal carcinomas [18]. Similar to the caspase cleaved soluble extracellular CK18 fragment, a serum biomarker for tumor cell apoptosis [22], intracellular intact CK18 appears to be a significant tumor biomarker with clinical utilities. However, characterization of tumor biomarker CK18 and the clinical significance of its expression in CTC have not been reported. Besides being a “tumor biomarker”, CK18, the acidic low molecular weight type I protein which always complexes with its basic high molecular weight counterpart type II CK8 (CK8/18), is also regarded as an “epithelial marker” for detection of CTCs [18].

Current CTC identification strategies mainly rely on confirmatory immunostaining of the “epithelial marker” CK8/18/19 in tumor cells. However, it has been recognized that during progression of EMT, down-regulation of EpCAM and CK is part of an oncogenic pathway that increases tumor invasiveness and metastatic potential [8, 9, 14, 23]. It has been reported that ectopic expression of vimentin and loss of CK, indicating EMT, in 2, 517 breast cancer patient samples was associated with a higher tumor grade and mitotic index[23]. Failure to detect CTCs or existence of “invisible” CTCs due to down-regulation or absence of CK [24] has been published [9, 19]. Moreover, tumor cells disseminated in cerebrospinal fluid (CSF) of extremely high-mortality glioma patients, who account for 70% of 22,500 annual new cases of brain tumors in the United States [25–27], do not express both EpCAM and CK. It is therefore imperative to develop an alternative strategy aside from CK staining alone, regardless of the type and stage of cancer, to effectively identify CTCs.

In this study, we developed a novel strategy integrating subtraction enrichment and immunostaining-FISH (SE-i•FISH^®^), which enables effective depletion of WBCs and non-hemolytic removal of RBCs, to establish an expeditious detection of non-hypotonic damaged and non-hematopoietic aneuploid CTCs regardless of CK or EpCAM expression and size variation ranging from similar or smaller than WBCs up to larger tumor cells [5, 28, 29]. Using this approach, we were able to efficiently detect, isolate, and characterize heterogeneous subpopulations of CTC, circulating tumor microemboli (CTM) and disseminated tumor cells (DTCs) derived from diverse types of solid tumor including lung, glioma, melanoma, osteosarcoma, pheochromocytoma, parathyroid, esophageal, breast, pancreatic, gastric, colon, cervical, ovarian, bladder, renal cell and hepatocellular carcinomas in mouse or patient's peripheral blood, bone marrow, cerebrospinal fluid, urine, malignant pleural effusion or ascites with high sensitivity and specificity despite numerous mesothelial cells. Those enriched viable and non-antibody perturbed native tumor cells are suitable for primary cell culture and additional downstream analyses. Studies performed by SE-i•FISH^®^ showed that 92 and 87% of lung and esophageal cancer patients respectively had detectable CTCs, and 90.5% of the confirmed advanced gastric cancer patients were CTC positive, compared with 54.8% detected by *CellSearch* from the same population of patients. In addition, comprehensive identification and characterization of highly heterogeneous subpopulations of CTC/DTC, enable classification of those neoplastic cells into diverse subtypes by *in situ* phenotyping of numbers of the desired tumor biomarkers and karyotyping of chromosome ploidy in CTCs/DTCs (*in situ* PK CTC or DTC). Illustration of the CTC/DTC subtypes possessing distinct clinic significance[30] will help guide more specific and significant genotypic, proteomic and functional analyses performed on the targeted single tumor cell [31, 32].

## RESULTS

### Subtraction enrichment of cancer cells with diverse EpCAM expression

**Figure 1:**
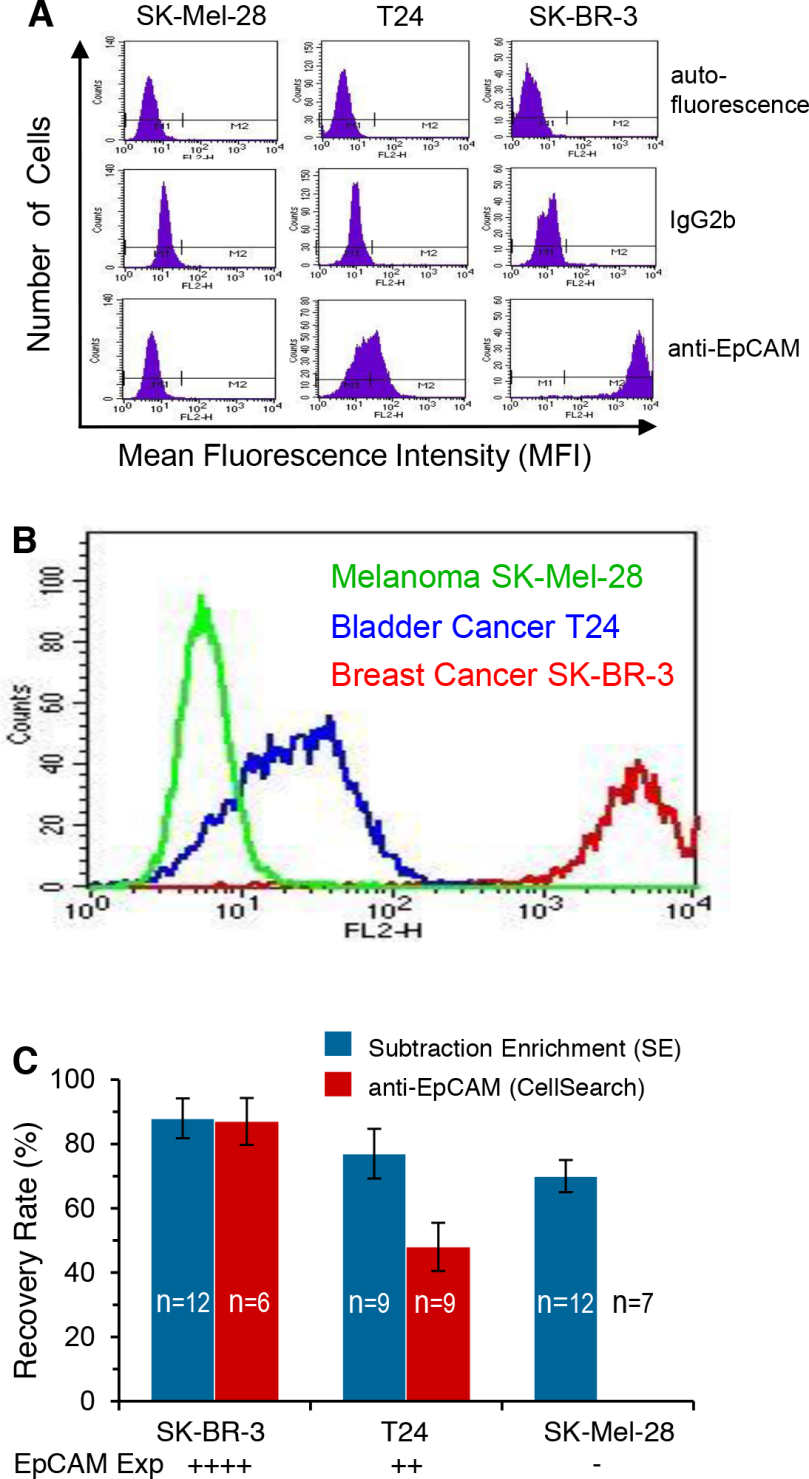
Characterization of EpCAM expression on tumor cells and comparison of isolation efficiency between subtraction enrichment (SE) vs anti-EpCAM capture strategy **A.** Compared to cell autofluorescence (auto) and the negative control of isotype IgG2b, PE-anti-EpCAM does not bind to the melanoma cell line SK-Mel-28, but does bind to the bladder cancer T24 and the breast cancer SK-BR-3 cell lines. **B.** Overlay of binding histograms demonstrates very strong binding of anti-EpCAM to breast (red) and medium strength binding to bladder cancer cells (blue), but no binding to melanoma cells (green). **C.** Results of recovery rate of the 2 different methodologies are demonstrated in 2 different colors. Eighty eight and 87% of the spiked SK-BR-3 breast cancer cells with high EpCAM expression (++++) are recovered by both SE (blue) and anti-EpCAM capture (red) strategy, respectively. Seventy seven percent of T24 bladder cancer cells with intermediate EpCAM expression (++) are enriched by SE, whereas 48% of those cells are isolated by anti-EpCAM. None of the SK-Mel-28 melanoma cells that do not express EpCAM (−) is isolated by anti-EpCAM technique. However, 70% of melanoma cells are recovered by the non-EpCAM-dependent SE. Results (mean ± SD) represent the average of values obtained in the number of separate experiments indicated by n.

Expression of EpCAM on SK-BR-3 breast, T24 bladder and SK-Mel-28 melanoma cancer cells was characterized by flow cytometry. Results in Figure 1A showed that compared to the negative isotype control IgG2b, anti-EpCAM similarly did not bind to SK-Mel-28 cells, but did bind to T24 and SK-BR-3 cancer cells. As shown in Figure 1B, further comparative analysis performed on the overlayed binding histograms demonstrated that anti-EpCAM bound strongly to SK-BR-3, weakly to T24, but not to SK-Mel-28 cells, indicating that breast and bladder cancer cells had high and medium expression of EpCAM, respectively. Melanoma cells did not express EpCAM.

Well-characterized cell lines were selected to demonstrate the limitations of anti-EpCAM capture for isolation of tumor cells bearing different expression levels of EpCAM. Between 3–50 of the indicated tumor cells labeled with MitoTracker were spiked into 7.5 ml of human blood, and subsequently subjected to non-EpCAM-dependent SE or antibody capture methodology. As shown in Figure 1C, 88 and 87% of SK-BR-3 cells with high EpCAM expression were recovered by both SE and anti-EpCAM, respectively. However, the recovery rate of anti-EpCAM for T24 cells with intermediate expression of EpCAM was reduced to 48%, while the recovery rate with SE was maintained as high as 77%. In the case of SK-Mel-28 melanoma cells that do not express EpCAM, the recovery rate with SE was 70%, whereas no cell was isolated with anti-EpCAM strategy.

### *In situ* Comprehensive phenotypic and karyotypic identification and characterization of CTCs and DTCs enriched from cancer patients’ and murine peripheral blood or malignant pleural effusion

**Figure 2:**
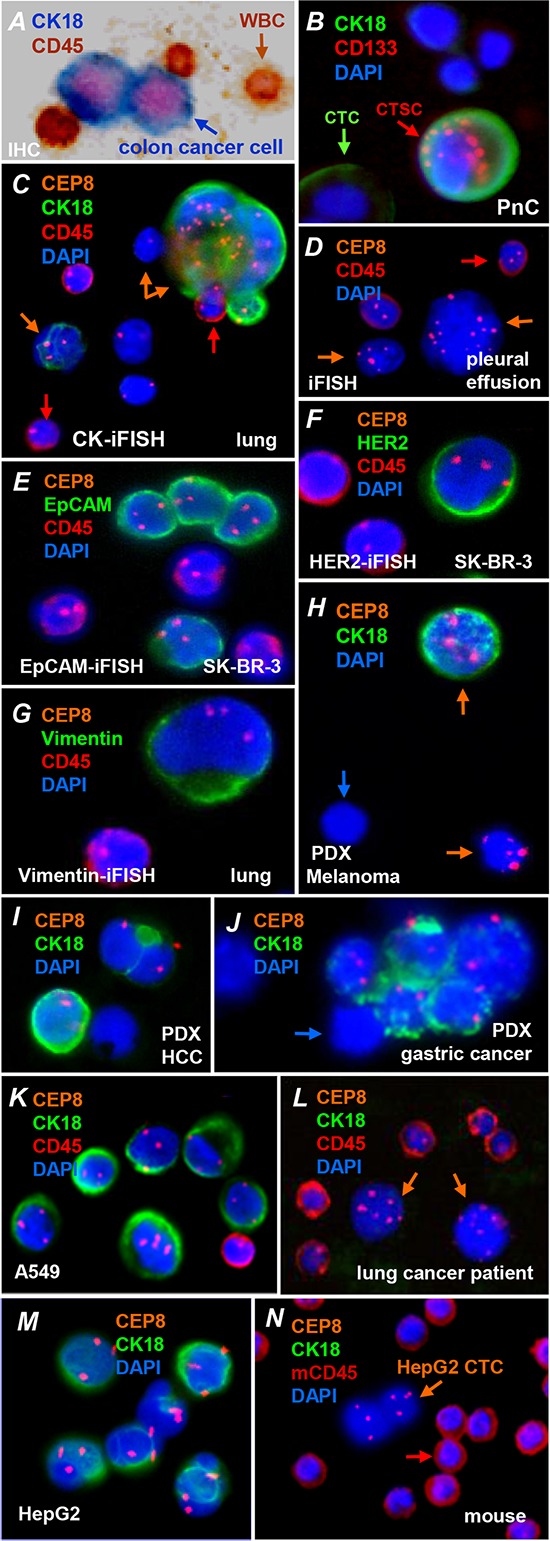
*In situ* Phenotyping and karyotyping CTCs and DTCs enriched from cancer patients or mice bearing human tumors **A.** Colon cancer cells (SW480) enriched from blood by SE are identified by immunohistochemistry staining and visualized under a bright field microscope. CK18 and nuclei of tumor cells are stained in blue and pink, respectively. WBCs show color of brown. **B.** A circulating tumor stem cell (CTSC) (CK18+, CD133+, red arrow) and CTC (CK18+, CD133-, green arrow) enriched from the blood of a pancreatic cancer patient are identified by immunofluorescent staining. **C.** Non-hematopoietic heteroploid lung cancer CTCs (orange arrows) enriched from a NSCLC lung cancer patient's blood sample are identified by CK18-iFISH, and respectively show strong, medium and undetectable CK18 expression. A large multiploid CTC with strong positive CK18, and a small triploid CTC with medium positive CK18 as well as a small monoploid CTC with undetectable CK18 expression are observed. WBCs are indicated by red arrows. **D.** Disseminated tumor cells enriched from the malignant pleural effusion of a patient with pancreatic cancer metastasizing to lung are identified by iFISH and characterized as non-hematopoietic heteroploid cells (CD45-, iFISH+, orange arrows). One of the tumor cells is similar in size to that of a WBC (red arrow). **E.** EpCAM-iFISH shows that enriched aneuploid breast cancer cells SK-BR-3 EpCAM+, iFISH+, and CD45-. **F.** An enriched triploid non-hematopoietic breast cancer cell (SK-BR-3) with distinct expression of HER2 is demonstrated by HER2-iFISH. **G.** Expression of Vimentin in an enriched non-hematopoietic NSCLC patient CTC is revealed by Vimentin-iFISH. **H.** Human melanoma CTCs (orange arrows) enriched from a metastatic mPDX mouse blood sample are identified by CK18-iFISH; they have either detectable (iFISH+, CK18+), or undetectable CK18 (iFISH+, CK18-) which is similar in size to a mouse WBC (blue arrow). Human CEP8 FISH probe doesn't hybridize to mouse WBC's chromosomes. **I.** Human HCC CTCs in a mPDX mouse show detectable CK18 with either homogeneous or polar distribution in cells (iFISH+, CK18+). **J.** Human gastric carcinoma CTCs enriched from the Cisplatin treated mPDX mouse model have both very strong and extremely weak CK18 expressing tumor cells in the enriched circulating tumor microemboli (CTM), and majority of those cells show a similar size to that of murine WBC (blue arrow). **K.** All aneuploid A549 lung cancer cells enriched from blood are CK18+ (green) (iFISH+, CK18+). **L.** Neither of 2 lung cancer patient CTCs shows detectable CK18 (iFISH+, CK18-, orange arrows). **M.** All human HepG2 HCC cells are aneuploid and CK18+ (iFISH+, CK18+). **N.** An identified CTM consisting of 2 heteroploid HepG2 CTCs in a mouse tumor model established with the same HepG2 cell line cells does not have detectable CK18 (iFISH+, CK18-, mouse CD45-, orange arrow). Murine WBCs identified by anti-mouse CD45 (mCD45) are indicated by a red arrow.

In Figure 2A, an immuno-histochemical (IHC) technique to stain CK18 (blue) identifies colon cancer cells SW480 enriched from blood. Blue color of the tumor cells with pink nuclei clearly distinguish them from the brown color of WBCs. Figure 2B shows a circulating tumor stem cell (CTSC) and a CTC enriched from peripheral blood of a pancreatic cancer patient. Of 2 CK18+ pancreatic cancer CTCs, one stained positively for both CK18+ and CD133+, potentially representing a CTSC [33]. Results of *in situ* phenotyping and karyotyping CTCs (*in situ* PK CTC) in Figure 2C reveals non-hematopoietic heteroploid CTCs identified by CK18-iFISH in a non-small cell lung cancer (NSCLC) patient's blood. A large strong CK18 positive multiploid CTC and 2 small “budding” CTCs are observed. The CK18+ budding CTC is diploid, whereas the CK18- budding CTC is monoploid. A medium CK18 positive triploid CTC in smaller size is also demonstrated. Two remaining CK18- diploid cells are not identified as CTC in this study. In Figure 2D, disseminated tumor cells enriched from the malignant pleural effusion of a patient with pancreatic cancer metastasizing to lung is shown. Cancer cells are identified as non-hematopoietic (CD45-) and polyploid by iFISH. One of the tumor cells is similar in size to a WBC.

To further expand investigation of expression of tumor biomarkers other than CK18 on CTCs by iFISH, co-immunofluorescent staining of several other tumor biomarkers in addition to CD45 was simultaneously performed on the same iFISH sample. As revealed in Figures 2E, EpCAM-iFISH demonstrates heteroploid chromosome 8 and strong EpCAM expression in enriched breast cancer cells SK-BR-3. Shown in Figure 2F, a triploid breast cancer cell SK-BR-3 enriched from blood has a strong expression of HER2 demonstrated by HER2-iFISH. Figure 2G shows a non-hematopoietic (CD45-) CTC enriched from a NSCLC patient has triploid chromosome 8 and visible EMT marker vimentin.

Similarly, we were able to demonstrate the authentic morphology of CTCs enriched from murine blood in patient-derived xenograft (PDX) mouse models with high metastasizing potential (mPDX). Figure 2H demonstrates 2 CTCs isolated from a melanoma - PDX model. Unlike the large triploid CK18+ CTC, another pentaploid CK18- melanoma CTC is similar in size to a mouse WBC. Figure 2I demonstrates human hepatocellular carcinoma (HCC) CTCs in a mPDX mouse. Both CTCs are CK18+ showing either homogeneous or a polar distribution pattern. Human gastric cancer CTCs in the form of a circulating tumor microemboli (CTM) enriched from the chemotherapeutic agent Cisplatin treated mPDX mouse model are demonstrated in Figure 2J. Highly heterogeneous expression of CK18 in individual CTCs among the CTM is demonstrated. Some CTCs have high CK18 expression, however, extremely low expression of CK18 is revealed in the remaining CTCs. Majority of those patient tumor cells in CTM have a size similar to that of mouse WBC.

### Absence of CK18 expression in tumor cell line derived CTCs but not in tumor cell line cells

In view of the fact that significant populations of CTCs without detectable CK18 are present in both patients and PDX mouse models of different types of cancer, expression of CK18 was examined by CK18-iFISH in both tumor cell lines and enriched CTCs to rule out the possibility that undetectability or absence of CK18 in CTCs is a consequence of the CK18-iFISH methodology.

As shown in Figure 2K, all aneuploid A549 lung cancer cells enriched in a blood sample from a healthy donor had detectable CK18. However, 2 of the multiploid lung cancer CTCs enriched from a lung cancer patient are CK18- (Figure 2L). Figure 2M shows that all of HCC HepG2 cells are heteroploid and CK18+, whereas a cluster of 2 heteroploid HepG2 CTCs in a CTM enriched from the blood of a mouse tumor model which was established with the exact same HepG2 cell line reveals non-detectable CK18 (Figure 2N). Human CEP8 did not hybridize to mouse WBC's chromosomes.

Our results indicate that the absence of CK18 in CTCs was not a consequence of the CK18-iFISH methodology itself, which is in agreement with the concept that tumor cells may regulate CK18 expression under certain circumstances, and such post-translational modulation of CK18 protein in CTCs revealed and quantified by phenotypic immunostaining is of particular biological and clinical significance.

### Validation of SE-i•FISH^®^

**Figure 3:**
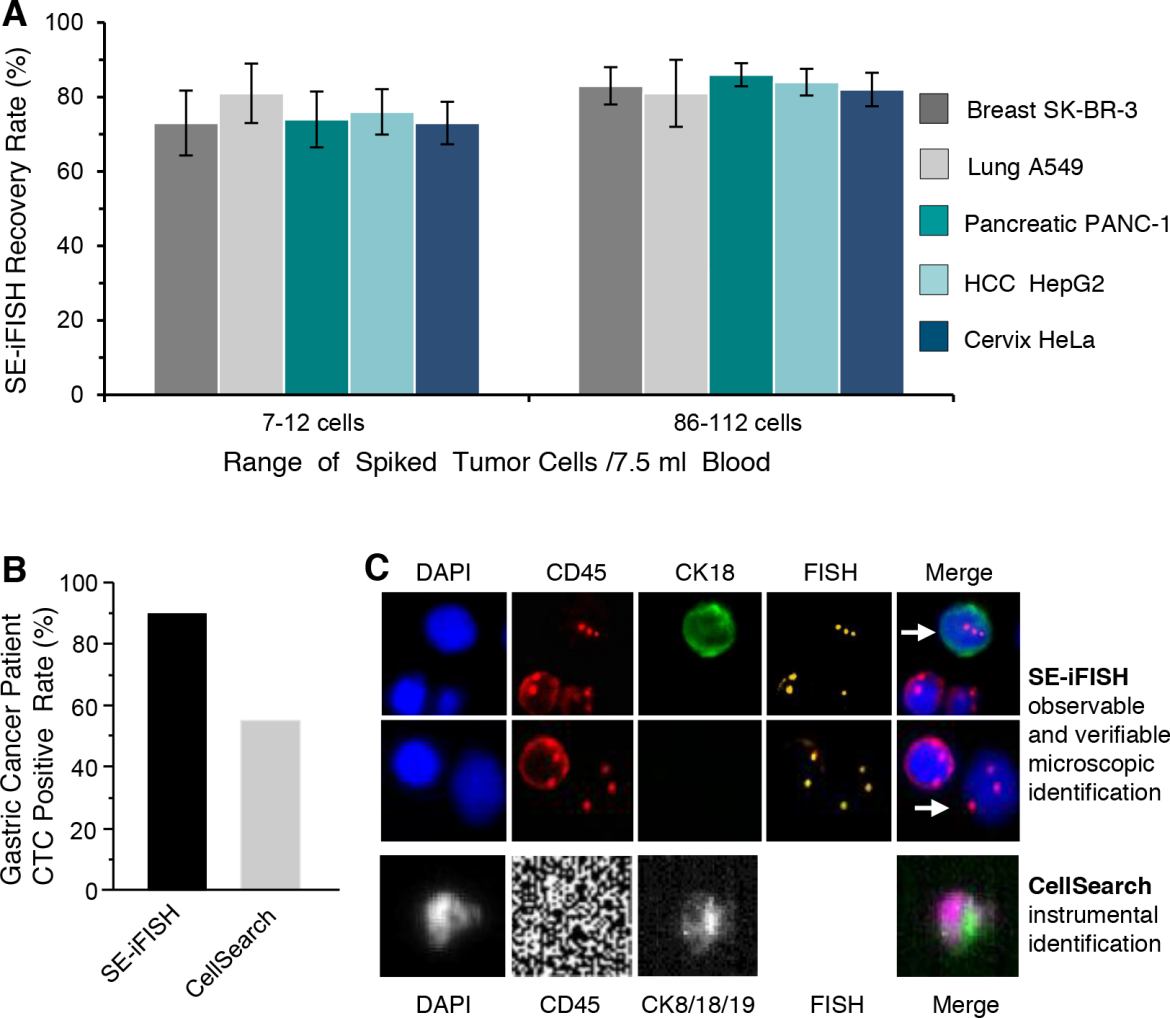
Validation of SE-iFISH **A.** For the group of low number of 7–12 spiked cells, recovery rate is 73% (breast), 81% (lung), 74% (pancreatic), 76% (HCC) and 73% (cervix), respectively. When high number of 86–112 of different tumor cells are spiked, 83% (breast), 81% (lung), 86% (pancreatic), 84% (HCC) and 82% (cervix) cancer cells are recovered by SE-iFISH. Results (mean ± SD) represent the average of values obtained in 3 separate experiments. **B.** Clinical validation of SE-iFISH on the advanced gastric cancer patients. Microscopically observed and verified SE-iFISH results show that 90.5% of patients have detectable non-hematopoietic aneuploid CTCs in 7.5 ml of blood, whereas CellSearch instrument detection demonstrates a positive rate of 54.8% for the same population of patients. **C.** Gastric cancer cells identified by iFISH (white arrow) are CK18+/triploid or CK18-/triploid. WBC shows a red ring of CD45 positive staining. Fluorescent image of cancer cells identified by iFISH is microscopically observable for verification. Gastric cancer CTC image acquired by CellSearch without available FISH identification is demonstrated.

To validate the efficiency of recovery by SE-iFISH, low (7–12) or high (86–112) numbers of breast, lung, pancreatic, HCC and cervical cancer cell line cells were spiked into 7.5 ml blood, followed by SE-iFISH identification. Recovered cells were enumerated by CK18 staining which was confirmed by FISH of chromosome 8. Results of Figure 3A demonstrate stable recovery of the indicated cancer cell line cells, showing 73–81% and 81–86% recovery for the cancer cells spiked at low and high numbers, respectively.

Further clinical validation was performed by comparison of SE-iFISH *vs CellSearch* on 7.5 ml of blood collected from 29 advanced gastric cancer patients. As shown in Figure 3B, the positive CTC detection rate was 90.5% for SE-iFISH and 54.8% for *CellSearch*. Figure 3C illustrates microscopically verified triploid CK18+ or CK18- gastric cancer cells identified by iFISH. An image of a gastric cancer CTC detected by the *CellSearch* instrument without available FISH characterization is also revealed [30].

### Analysis of cancer patient CTCs and their subtypes

Lung and esophageal cancer patient blood samples were subjected to subtraction enrichment (SE), followed by CK18-iFISH analysis. Non-hematopoietic heteroploid CTCs without detectable CK18 (CD45-, CK18-, FISH+, DAPI+), and those with strong CK18 positive (CD45-, CK18+, FISH+, DAPI+) observed and verified by means of a fluorescence microscope were enumerated, respectively. Detailed clinical information of lung and esophageal cancer patients and enumeration of CTC subtypes as well as total number are described in Tables 1 and 2, respectively.

**Table 1:** CTCs and their subtypes of lung cancer patients

Patient Code	Staging	Type	CK18	Ploidy of Chromosome 8					CTC Count	
				1	2	3	4	≥5	CK Subtype	Total
1	IB	ADC	−			4			4	**4**
2	IB	ADC							0	**0**
3	IIA	SCC	−			1		2	3	**4**
			+		1				1	
4	IIA	ADC	–	1					1	**1**
5	IIB	ADC	−		2*			10	10	**10**
6	IIIA	ADC	−			12		16	28	**28**
7	IIIA	ADC	−			6		2	8	**14**
			+				1		1	
			++	1	1	3			5	
8	IIIA	SCC	−						0	**0**
9	IIIA	ADC	−	1		43	2	29	75	**75**
10	IIIA	SCC	−			2		6	8	**8**
11	IIIA	ADC	−			8		5	13	**19**
			+			6			6	
12	IIIB	ADC	−	2	1*	5		13	20	**20**
13	IIIB	ADC	−			53		76	129	**129**
14	IIIB	SCC	−			23		3	26	**35**
			+				3		3	
			++			6			6	
15	IIIB	SCC	−					3	3	**3**
16	IIIB	ADC	−			11		8	19	**19**
17	IV	SCC	−			16		36	52	**52**
18	IV	ADC	−			12		19	31	**39**
			++		4	4			8	
19	IV	LCLC	−	2		3	4	3	12	**12**
20	IV	ADC	−					1	1	**1**
21	IV	ADC	−			3		5	8	**20**
			+		2				2	
			++					10	10	
22	IV	SCC	−			2	2	4	8	**8**
23	IV	ADC	+			6		18	24	**32**
			++	2	1	4	1		8	
24	IV	ADC	−			1	2	2	5	**5**
25	IV	SCLC	−	4	2	22		36	64	**64**
26	IV	SCLC	−					9	9	**21**
			++			5	1	6	12	

The clinical information and status of patients and CTCs, including phenotyping of CK18 expression and karyotyping of chromosome 8 ploidy as well as number of CTCs.

CK18-, no detectable CK18; C18K+, weak detectable CK18; CK18++, strong detectable CK18

* CK18 negative diploid non-hematopoietic cells were not counted as CTC in this study

**Table 2:** CTC and their subtypes of esophageal cancer patients

Patient Code	Staging	Type	CK18	Ploidy of Chromosome 8					CTC Count	
				1	2	3	4	≥5	CK Subtype	Total
1	IA	SCC	−					3	3	**3**
2	IA	SCC	−	1				1	2	**2**
3	IB	ADC	−							**0**
4	IIA	SCC	−		4*	3			3	**3**
5	IIIA	SCC	−			3		2	5	**5**
6	IIIA	SCC	−		2*	6	2	5	13	**19**
			++		4			2	6	
7	IIIA	SCC								**0**
8	IIIB	SCC	−			14		1	15	**34**
			+		2				2	
			++	3	1	4	2	7	17	
9	IIIB	SCC	++					12	12	**12**
10	IIIB	SCC	−					6	6	**6**
11	IIIB	ADC	−			8		10	18	**28**
			++		1	5	1	3	10	
12	IIIB	SCC	−			3		8	11	**11**
13	IV	ADC	−		1*	9	2	12	23	**23**
14	IV	SCC	−	1		2		4	7	**7**
15	IV	SCC	−			3		5	8	**12**
			++		2	2			4	

The clinical information and status of esophageal cancer patients and CTCs, including phenotyping of CK18 expression and karyotyping of chromosome 8 ploidy as well as number of CTCs.

CK18-, no detectable CK18; C18K+, weak detectable CK18; CK18++, strong detectable CK18

* CK18 negative diploid non-hematopoietic cells were not counted as CTC in this study

**Figure 4:**
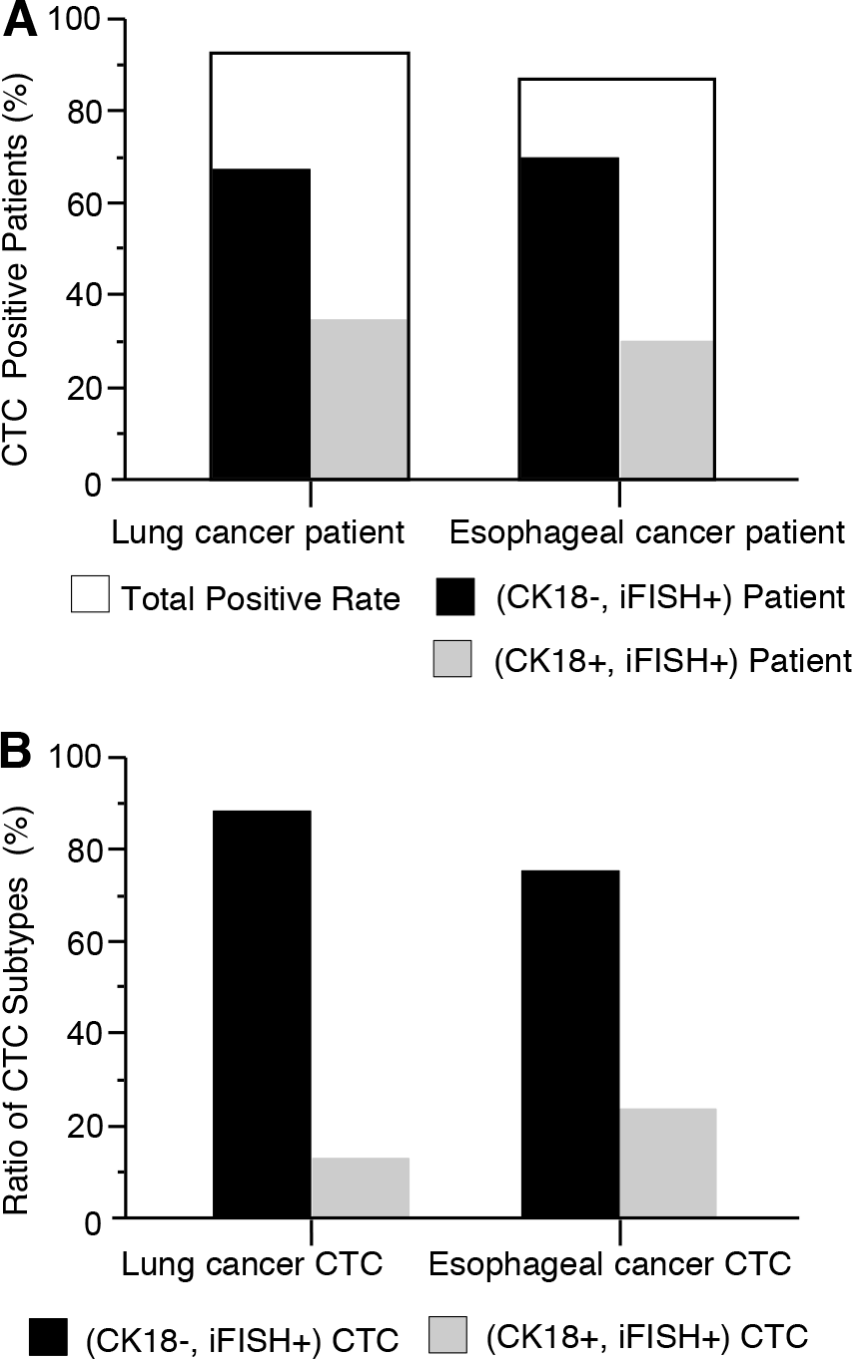
Detection of CTCs in cancer patients by non-EpCAM dependent SE-iFISH **A.** Detection of CTCs in lung and esophageal cancer patients. The net CTC positive rate including both observable strong CK18+ and CK18- subtypes is 92% (24/26 patients) for lung cancer, and 87% (13/15) for esophageal cancer patients (non-color column), respectively. Among 24 CTC-positive lung cancer patients, 67% (16/24, black) are CK18- (CK18-, iFISH+), remaining 33% patients (8/24, grey) have CK18+ CTCs (CK18+, iFISH+). For 13 esophageal cancer patients who are CTC positive, 69% (9/13) are CK18- (black) (CK18-, iFISH+), whereas 31% (4/13) patients are CK18+ in at least some of the CTCs (CK18+, iFISH+) (grey). **B.** Analysis of CK18 expression in CTCs. In a total of 623 lung cancer CTCs, 86% (537/623, black) are CK18-; the remaining 14% (86/623, grey) have detectable CK18. For the total of 165 esophageal CTCs, 76% (126/165, black) have no detectable CK18; the other 24% (39/165, grey) are CK18+.

As shown in Figure 4A, the overall positivity of CTCs isolated from lung cancer patients was 92% (24/26 patients, non-color column) with a range of 1–129 CTCs/7.5 ml blood. Further *in situ* PK CTC analysis indicated that among the 24 CTC positive patients, 16 (16/24, 67%, black) were CK18-/iFISH+, showing no visible CK18 in any of the detected CTCs. The remaining 8 of CK18+/iFISH+ patients (8/24, 33%, grey) had a mixed population of CTCs, of which both CK18+ and CK18- CTCs were found. For the 15 esophageal cancer patients, the overall CTC positivity was 87% (13/15, non-color column) with a range of 2–34 CTCs/7.5 ml blood; 9 of them were CK18-/iFISH+ patients (9/13, 69%, black), and the remaining 4 were CK18+/iFISH+ patients (4/13, 31%, grey).

As revealed in Figure 4B, further analysis of CK18 expression on lung cancer CTCs indicated that in a total of 623 detected CTCs, 86% cells (537/623, black) were CK18-, while 14% (86/623, grey) had detectable CK18+. Of the 165 CTCs isolated from patients with esophageal cancer, 76% (126/165, black) were CK18-, whereas the remaining 24% (39/165, grey) were CK18+. These results suggest that immunofluorescent staining of CK18 alone to identify CTCs may result in a significant false negative detection.

Additional analysis of subtypes of CTC classified by both chromosome 8 ploidy and CK18 expression indicated that CTC could be detected in all stages (I-IV) of lung and esophageal cancer patients. As shown in Table 3, about half of total detected lung CTCs (51.5%) were penta- or multiploid (five or more copies) chromosome 8 with CK18 positivity of 5.5% and negativity of 46%, respectively. The second to the largest population of lung CTC subtypes were triploid chromosome 8 (41.9%), which included 5.5% CK18+ and 36.4% CK18- cells. A similar observation was obtained on esophageal cancer CTCs. Forty nine% were penta- or multiploid (five or more copies) chromosome 8 with CK18 positivity of 7.3% and negativity of 41.8%, respectively. About 38% of esophageal cancer CTCs were triploid chromosome 8, and among this population, 6.7% were CK18+, and 30.9% were CK18-. For both prior to treatment lung and esophageal cancer patients, triploid and penta- or multiploid (five or more copies) chromosome 8/CK18- cells constituted the largest population of CTC subtype in this study.

**Table 3:** Phenotypic and karyotypic analysis of CTC subtypes

CK18 Expression	Ploidy of Chromosome 8				
	1	2	3	4	≥5
**Lung cancer CTC**					
CK+ subtype	0.5% (3/623)	1.8% (11/623)	5.5% (34/623)	1% (6/623)	5.5% (34/623)
CK- subtype	1.6% (10/623)	0.5% (3/626)*	36.4% (227/623)	1.6% (10/623)	46% (288/623)
Sum	2.1%	1.8%	41.9%	2.6%	51.5%
**Esophageal cancer CTC**					
CK+ subtype	1.8% (3/165)	6.1% (10/165)	6.7% (11/165)	1.8% (3/165)	7.3% (12/165)
CK- subtype	1.2% (2/165)	4% (7/172)*	30.9% (51/165)	2.4% (4/165)	41.8% (69/165)
Sum	3.0%	6.1%	37.6%	4.2%	49.1%

Data are given as percentage (number/total). CTCs with five or more copies of chromosome 8 without CK18 expression constitute the largest population of lung and esophageal cancer CTCs.

* CK18 negative diploid non-hematopoietic cells were not counted as the confirmatory CTCs in this study

Among 21 healthy donors, 1 subject was found to have 1 CK18- monoploid non-hematopoietic cell with unknown significance.

### Detection of disseminated heteroploid glioma tumor cells in cerebrospinal fluid

**Figure 5:**
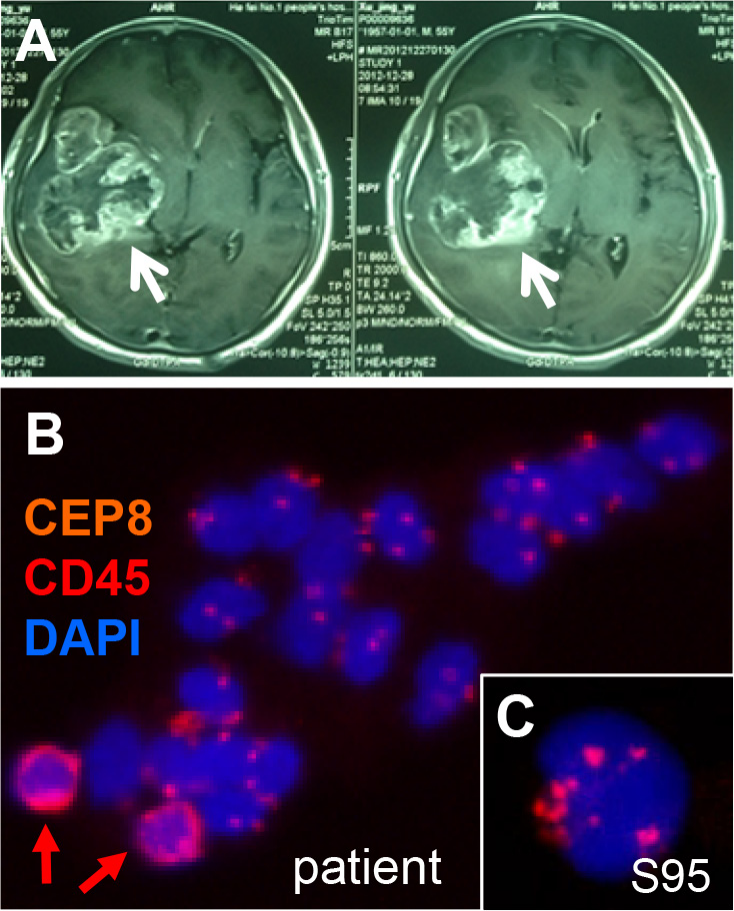
Detection of heteroploid glioma tumor cells disseminated in cerebrospinal fluid of a malignant glioma patient **A.** MRI scans of the glioma patient. The right temporal glioblastoma with obvious enhancement and necrosis is indicated by arrow. **B.** A cluster of malignant heteroploid glioma tumor cells (CTM) enriched from cerebrospinal fluid of the same patient is shown. WBCs are indicated by red arrows. **C.** Image of the cultured primary glioblastoma cells S95 shows heteroploid chromosome 8.

A 55-year-old male patient was diagnosed glioblastoma, WHO IV (Huashan Hospital, Shanghai, China). The malignant tumor in patient's temporal was revealed by Magnetic Resonance Imaging (MRI) scans (Figure 5A). Glioma cells were enriched from CSF of the same patient, followed by iFISH identification. Figure 5B showed a tumor microemboli consisting of numbers of non-hematopoietic heteroploid glioma cells disseminated in CSF. Hematopoietic WBCs were identified by CD45 staining. Cultured aneuploid primary glioblastoma cells S95 (Huashan Hospital) were identified and characterized by iFISH as shown in Figure 5C.

## DISCUSSION

Evidence for the clinical significance of CTCs has been published elsewhere. In addition, EGFR mutation analysis performed on lung cancer CTCs was reported to be more sensitive than conventional serum nucleic acid analysis [34]. However, current EpCAM-dependent antibody capture and CK-dependent identification strategies are restricted and biased to the only both CK and EpCAM positive CTCs [8, 14], thus having significant limitations particularly in their ability to capture and identify CTCs shed from several different types of cancer including lung (NSCLC), melanoma, glioma, renal cell and pancreatic cancers, etc. [8,9,14]. Importantly, the complex heterogeneity of CTCs has not been and cannot be recognized by such approaches.

To date, efforts to improve CTC detection have focused on either isolation or identification, respectively. In view of the failure to detect a significant population of both “uncapturable” and “invisible” CTCs due to inherent drawbacks of current CTC strategies, we extended our previous efforts [35] in the present study to develop an integrated subtraction enrichment (SE)-immunostaining FISH (i•FISH^®^) platform for both efficient enrichment and identification of circulating tumor microemboli (CTM) and CTCs as well DTCs derived from various solid tumors in both patients and mouse models bearing human tumors.

The most recognized “negative enrichment” applies anti-CD45 antibody to deplete WBCs [29, 36], which may have significant amounts of remained WBCs (at least 10 thousands cells) as well as post-enrichment residual blood components, resulting in markedly interfering subsequent CTC identification performed by FISH or iFISH. Moreover, both deleterious hypotonic damage and loss of CTCs following hemolysis of RBC has been reported [29, 36, 37]. The SE strategy described in this study takes advantage of the coated immunomagnetic beads conjugated to a cocktail of anti-multiple WBC surface markers, and the unique non-hematopoietic cell separation matrix to remove RBCs without hypotonic hemolysis, and efficiently deplete WBCs on the order of 4–5 logs. Minimal non-specific adhesion of epithelial tumor cells to the immunomagnetic beads with special coating is maintained during enrichment. Rapidly enriched, non-hematopoietic and non-antibody perturbed CTCs and DTCs which are free of hypotonic injury are suitable for subsequent primary tumor cell culture (our unpublished results) and a series of analyses performed on either pooled or single tumor cell [32].

Aneuploidy of chromosome 8 examined by CEP8-FISH has been reported on neoplastic cells from tissues of several types of tumors including lung [38], esophageal [39], pancreatic [40], gastric [41], colon [42], bladder [43] and hepatocellular [44] carcinomas, etc. However, attempts to apply the combined immunofluorescent staining and FISH to identify CTCs were complicated due to inherent bio-complicacy of hematopoietic WBCs and non-hematopoietic neoplastic cells [45, 46] as well as lengthy conventional FISH experimental procedure. In this study, we developed a novel *in situ* strategy combining FDA-approved karyotypic CEP8-FISH and simultaneous phenotypic immunofluorescent staining of tumor biomarkers with either intracellular or extracellular antigenic epitopes (such as HER2 [30], CK, EpCAM, CD133, Vimentin, CD44V6, etc.) as well as CD45 on the identical cells to successfully identify non-hematopoietic, heteroploid tumor cells. Such *in situ* i•FISH^®^ strategy has been confirmed to effectively identify and characterize various neoplastic cells derived from solid tumors, including CTCs enriched from patients’ peripheral blood, and DTCs enriched from cerebrospinal fluid (CSF), bone marrow, urine, malignant pleural effusions and ascites. Moreover, in contrast to conventional time-consuming protocols for FISH methodology alone, *i.e*., more than 20 hours, the entire i•FISH^®^ procedure including antibody staining is accomplished in as short as 3–4 hours.

*In situ* Phenotyping and karyotyping CTCs (*in situ* PK CTC) by i•FISH^®^ demonstrated the majority of CTCs identified in patients (Figures 2C and 2L, 4) or the most cancer cell line CTCs in mouse blood in this study (Figure 2N) had no detectable or visible CK18 or PanCK including CK4, 5, 6, 8. 10, 13 and 18 (data not shown), however, the same procedure showed strong CK18 staining in lung and HCC cancer cell line cells (Figure 2K and 2M) and in all of more than 20 of epithelial tumor cell lines including breast, pancreatic and colon cancers, etc. (data not shown) as well as in majority of CTCs detected in metastatic PDX (mPDX) mice [47], indicating that loss of detectable CK18 in CTCs is an unlikely consequence of the i•FISH^®^ methodology. Our results suggest that CK18 has significant limitations as an “epithelial marker” for CTC detection. It has been recently recognized that CK18 is a “tumor biomarker” with distinct clinical significance in cancer cells [18]. Post-translational down-regulation of intracellular CK18 protein revealed by phenotypic immunostaining was found to promote cell migration [20] and progression of breast [19] and colon cancers [48], whereas up-regulated CK18 protein was reported to correlate to poor differentiation and advanced stage in lung [49], renal cell [50], oral cavity [51] and esophageal squamous cell [52] carcinomas. Our on-going clinical studies performed on gastric, pancreatic, lung and cholangiocellular carcinoma patients indicated that CTCs with visible strong CK18 expression revealed by i•FISH^®^ seemed to correlate to patients’ rapid tumor progression and high mortality even at TNM early stage. Elucidation of potential mechanisms in addition to EMT [9] accounting for regulation of CK18 expression in CTCs and its subsequent correlations with clinical outcomes requires further investigation.

CTCs can be classified into different subtypes based on *in situ* phenotyping of CK or other tumor biomarker expression and karyotyping of chromosomal ploidy, i.e. CK18+ and CK18- subtypes in this study, each with 1 to ≥ 5 copies of chromosome 8 or any other chromosome(s), respectively. Our on-going preliminary multi-center studies indicate that in addition to lung, esophageal and gastric carcinomas, there is a high frequency of CTC subtypes with diversified CK18 expression in several types of cancer including renal cell, HCC, ovarian, colorectal, and pancreatic cancers, etc. (unpublished results). Analysis of CTC subtypes described in Tables 1 and 2 suggests that delineation of specific CTC subtypes (Table 3) and their correlation with clinical outcomes should be straight-forward in prospective clinical studies. Indeed, one of the anticipated clinical significance of CTC subtypes has been confirmed in our recent studies performed on the advanced gastric cancer patients, showing that among CK18 negative CTCs, trisomy in chromosome 8 CTC may possess intrinsic resistance to the chemotherapeutic drug cisplatin, compared to the tetra- and/or pentasomy subtype which developed the acquired cisplatin resistance [30]. Similar results from mPDX mice study were also recently published [47]. Mesenchymal status of those CK18- CTC possessing diverse chemosensitivities are currently under our investigation on large cohorts of patients and metastatic PDX (mPDX) mice extended from our previous study [47] by means of EpCAM and/or Vimentin-i•FISH^®^.

In conclusion, regardless of cellular heterogeneity, inherited down-regulation or absence of CK and EpCAM [6, 7] or other tumor cell surface molecules, we obtained efficient detection of both CTCs and DTCs from mice or patients with diverse types of cancer. Application of subtraction enrichment (SE) integrated with *in situ* PK CTC or DTC performed by i•FISH^®^ enables straight-forward specific comprehensive identification and characterization of non-hematopoietic heteroploid CTCs/DTCs and their subtypes. It is anticipated that SE-i•FISH^®^ or its combination with other CTC techniques will help guide and promote more specific and significant either pooled or single cell based genomic, protein and functional studies of tumor cells, and will also help establish polyclonal or potential monoclonal CTC/DTC or its subtype-derived “xenograft” (CDX) models [53]. Follow-up studies correlating clinical significance, such as prognosis, metastasis, drug resistance and cancer recurrence with CTC and DTC subtypes identified by a number of tumor biomarkers-i•FISH® in large cohorts of multi-cancer types of patients including malignant glioma (glioblastoma), are currently under active investigation.

## MATERIALS AND METHODS

### Patients and animals

Twenty six lung cancer patients including 16 ADC (2 IB, 1 IIA, 1 IIB, 4 IIIA, 3 IIIB, 5 IV), 7 SCC (1 IIA, 2 IIIA, 2 IIIB, 2 IV), 1 large cell lung cancer (LCLC) (IV), and 2 small cell lung cancer (SCLC) (IV), and 15 esophageal cancer patients including 3 adenocarcinoma (ADC) (1 staging IB, 1 IIIB, 1 IV) and 12 squamous cell carcinoma (SCC) (2 IA, 1 IIA, 3 IIIA, 4 IIIB, 2 IV), as well as 21 healthy donors were recruited in this study. All patients were newly diagnosed and untreated. All cancer diagnoses were confirmed by histopathological analysis.

Consent forms signed by all human subjects recruited to this study were approved by the Ethics Review Committees (ERC) of the Capital Medical University (CMU) Cancer Center, Beijing, and Huashan Hospital, Shanghai, China. The written informed consent forms were received from patients prior to inclusion in the study. The study was performed according to the Declaration of Helsinki Principles.

Animal related studies described in this paper were approved by the Ethics Review Committee (ERC) of CMUCC.

To avoid bias, blood sample collection, encoding, enrichment, SE-iFISH and result reading were blindly performed by different personnel. Decoding, analysis and evaluation of CTC subtypes correlating to patient clinical status were co-performed by cross-blinded physicians and research scientists.

### Examination of EpCAM expression on tumor cells by flow cytometry

Resuspended SK-BR-3 breast, T24 bladder, and SK-Mel-28 melanoma cancer cells were washed and resuspended in 1% BSA-PBS to achieve a cell concentration of 1 × 10^6^ cells/ml. Cell mixtures were incubated with PE conjugated anti-EpCAM (BD Biosciences, San Jose, CA, USA) or IgG2b as a negative control (0.5 μg/test) at room temperature for 15 min. Cells were washed and resuspended in 1 ml of 1% BSA in PBS containing 7-Aminoactionomycin D (7-AAD) (Life Technologies, Carlsbad, CA, USA), followed by flow cytometric analysis on the gated live cells.

### Subtraction enrichment (SE)

Experiment was performed according to the product manufacture's instruction (Cytelligen, San Diego, CA, USA). Briefly, 7.5 ml peripheral blood or 3 ml bone marrow were collected and centrifuged at 600 × g for 5 min. All sedimented cells were loaded on the top of 3 ml of non-hematopoietic cell separation matrix, followed by centrifugation at 400 × g for 5 min. Solutions above RBC were collected and incubated with 150 μl of anti-WBC and endothelial cell immunomagnetic beads for 15 min, followed by transferring to the top of the separation matrix. Samples were centrifuged at 400 × g for 5 min. Supernatants were collected and subjected to magnetic separation of beads. Bead-free solution was spun at 500 × g for 2 min. The resulting pellet containing rare cells was thoroughly mixed with 100 μl cell fixative, followed by application to the formatted and coated CTC slide (Cytelligen). Air dried samples are suitable for subsequent analyses, including immunohistochemistry (IHC) or immunofluorescent staining and iFISH described below.

Enrichment of glioma tumor cells from cerebrospinal fluid of malignant glioma or glioblastoma patients was described in product instruction (Cytelligen). Briefly, 10 ml of freshly collected CSF were thoroughly mixed with 1 ml of CSF preservative reagent (Cytelligen). Samples were centrifuged at 1050 × g for 3 mins, followed by discarding supernatant. Cell pellets were washed twice with 1x CSF solution (Cytelligen), and fixed on the coated CTC slides as described above.

Enrichment of CTC from mice was carried out according to the product manufacture's instruction (Cytelligen). Briefly, 50–200 μl of blood were collected from mice via retro-orbital bleeding, followed by immediately thorough mixing with the provided anti-coagulant suitable for mouse blood. Samples were subsequently subjected to enrichment performed with immuno-magnetic beads conjugated to anti-mouse leukocyte monoclonal antibody.

Coded blood sample collection, SE-iFISH and decoding were performed by different personnel in a blinded fashion.

### Immunohistochemistry staining of CTC

A mixture of monoclonal anti-CK18 (ImmunoBiosciences, Mukilteo, WA, USA) conjugated to FITC and monoclonal anti-CD45 labeled with digoxigenin (Roche Applied Sciences, Indianapolis, IN, USA) was incubated with cells fixed on the coated CTC slides at room temperature for 1 hr. After washing with PBS, samples were incubated with 150 μl of peroxidase (POD) conjugated monoclonal anti-digoxigenin and alkaline phosphatase (AP) labeled monoclonal anti-FITC at room temperature for 1 h. The color was developed by incubation with 3, 3′-diaminobenzidine (DAB) and AP Vector Blue working solution, and cell nuclei were stained with Vector Nuclear Fast Red according to the kit instruction (Vector Laboratories, Burlingame, CA, USA).

### Immunofluorescent staining of CTC

The procedure was performed similarly to that previously published [54, 55]. Briefly, cells were fixed on the coated CTC slides and incubated with 200 μl of monoclonal anti-CK18 conjugated to Alexa Fluor 488 and monoclonal anti-CD45 conjugated to Alexa Fluor 594 (Cytelligen) or CD133 conjugated to phycoerythrin (Miltenyi Biotech, San Diego, CA, USA) for 1 h in the dark. The concentration of all applied antibodies was adjusted to 2 μg/ml. Samples were washed with PBS, followed by mounting with 10 μl of mounting media containing DAPI (Vector Laboratories). Samples were subjected to image acquisition and analysis. CTC is defined as CK18 and/or CD133+, CD45- and DAPI+.

### CK18, EpCAM, HER2 or Vimentin-i•FISH^®^

Experiment was performed according to the product manufacture's instruction (Cytelligen). Briefly, samples on the coated CTC slides were subjected to Vysis Centromere Probe (CEP8) SpectrumOrange (Abbott Laboratories, Abbott Park, IL, USA) hybridization for 90 min using a S500 StatSpin ThermoBrite Slide Hybridization/Denaturation System (Abbott Molecular, Des Plaines, IL, USA), followed by incubation with Alexa Fluor 594 conjugated monoclonal anti-CD45 and Alexa Fluor 488 conjugated with monoclonal anti-CK18, EpCAM, HER2 or Vimentin (Cytelligen) as described above. Images of the identified tumor cells were collected using a fluorescence microscope (Nikon, Model Ni-U) equipped with a filter set (Omega Optical, Brattleboro, VT, USA) for DAPI (Cat. No. XF408), Alexa Flour 488 (Cat. No. XF421), Alexa Flour 594 (Cat. No. XF 414), and Spectrum Orange, TRITC (Cat. No. XF422). CTC is defined as DAPI+, CD45-, heteroploid CEP8 signal with or without visible CK18 or other tumor biomarkers.

### *CellSearch* detection

Experiment procedure was essentially similar to that previously described [1]. Briefly, blood samples were processed with CellPrep and *CellSearch* Epithelial Cell Kit, followed by staining with DAPI, allophycocyan conjugated anti-CD45 mAb and phycoerythrin conjugated anti-CKs 8, 18, 19 mAbs. Enumeration of positive tumor cells was performed by the CellSpotter Analyzer (Veridex, Raritan, NJ, USA). CTCs were identified as DAPI+, CKs+ and CD45-.
